# Tubal ligation and ovarian cancer risk in a large cohort: Substantial variation by histological type

**DOI:** 10.1002/ijc.29856

**Published:** 2015-10-08

**Authors:** Kezia Gaitskell, Jane Green, Kirstin Pirie, Gillian Reeves, Valerie Beral; on behalf of the Million Women Study Collaborators

**Affiliations:** ^1^ Cancer Epidemiology UnitUniversity of OxfordOxfordUnited Kingdom

**Keywords:** ovarian cancer, tubal ligation, sterilization, histological subtypes, histotype

## Abstract

Histopathological and molecular studies suggest that different histological subtypes (histotypes) of ovarian cancer have different aetiologies. Few studies have been large enough to explore reliably the effect of tubal ligation (sterilization), which has been associated with a reduced overall risk of ovarian cancer, on different tumour histotypes. In a prospective study of 1.1 million UK women without prior cancer or bilateral oophorectomy, 8,035 ovarian cancers occurred during mean follow‐up of 13.8 years. Using a Cox proportional hazards model, we estimated adjusted relative risks of ovarian cancer associated with tubal ligation. Overall, there was substantial heterogeneity in tumour risk associated with tubal ligation for the four main histotypes, serous, endometrioid, mucinous and clear cell (heterogeneity: *p* < 0.0001). For serous tumours, the most common histotype (*n* = 3,515), risks differed significantly between high‐grade (RR: 0.77, 95% CI: 0.67–0.89) and low‐grade tumours (RR: 1.13, 95% CI: 0.89–1.42); heterogeneity: *p* = 0.007. Relative risks were almost halved for endometrioid (*n* = 690, RR: 0.54, 95% CI: 0.43–0.69) and clear cell tumours (*n* = 401, RR: 0.55, 95% CI: 0.39–0.77), but there was no association between tubal ligation and mucinous tumours (*n* = 836, RR: 0.99, 95% CI: 0.84–1.18). For the main tumour histotypes we found little variation of risk by timing of tubal ligation. The significant differences by tumour histotype are unlikely to be due to confounding and are consistent with hypotheses that high‐grade and low‐grade serous tumours have different origins, and that some endometrioid and clear cell tumours might arise from cells and/or carcinogens travelling through the fallopian tubes.

## Abstract 2

**What's new?**

Tubal ligations can reduce ovarian cancer—but possibly only certain types. This large study reports on the risk of four main ovarian tumour histotypes following tubal ligation. They found that the procedure almost halved the risk of clear cell tumors and endometrioid tumours, but had little effect on mucinous tumours. For serous tumours, risk varied by grade: tubal ligation reduced the risk of high‐grade, but not low‐grade, serous tumours. These findings support the hypothesis that high‐ and low‐grade serous tumours have different causes, and that some ovarian tumours may arise from cells or carcinogens travelling through the fallopian tubes.

**Abbreviations**
BMI: body mass index
CI: confidence interval
FIGO: International Federation of Gynecology and Obstetrics
ICD: International Classification of Diseases
IQR: interquartile range
NCIN: National Cancer Intelligence Network
NHS: National Health Service
NHSCR: National Health Service central register
RR: relative risk
SD: standard deviation
WHO: World Health Organization

Ovarian cancer is one of the most common causes of cancer death amongst women in high income countries.^1^ There is increasing evidence that the different histological subtypes (histotypes) of ovarian cancer may have different origins, and show different molecular signatures.^2^, ^3^, ^4^, ^5^ The vast majority of ovarian cancers are epithelial tumours, of which the four most common histotypes are serous, mucinous, endometrioid and clear cell tumours.^6^ For serous tumours, it has been further suggested that they be divided into low‐grade serous tumours (consisting of serous borderline tumours and low‐grade serous carcinoma) and high‐grade serous carcinoma.

Meta‐analyses of observational epidemiological studies have consistently found that tubal ligation (also known as sterilization, in which the fallopian tubes are clipped, cut, or tied) is associated with an overall reduced risk of ovarian cancer. However, published findings by tumour histotype are inconsistent, except perhaps that most investigators have reported a reduced risk for endometrioid tumours.^7^, ^8^, ^9^ Epidemiological studies need to be sufficiently large to have enough power to detect reliably heterogeneity by tumour histotype. We therefore report here on the association observed between tubal ligation and incident ovarian cancer in a large cohort study of UK women.

## Material and Methods

### Study design, data collection and follow‐up

The Million Women Study is a prospective study of 1.3 million UK women, recruited in 1996–2001 *via* the UK National Health Service (NHS) Breast Screening Programme. At recruitment, women completed a questionnaire on socio‐demographic, reproductive, medical and lifestyle factors. The cohort is resurveyed approximately every 3 to 5 years. The study design and methods are described in detail elsewhere,^10^, ^11^ and questionnaires can be viewed online at http://www.millionwomenstudy.org.

All participants have been flagged on the NHS Central Register (NHSCR), so the study investigators are routinely notified of cancer registrations and deaths. The information provided includes the date of the event (cancer registration or death), together with the cancer site (coded using the 10th revision of the International Classification of Diseases, ICD‐10)^12^ and tumour morphology (coded using the second and third editions of the International Classification of Diseases for Oncology, ICD‐O).^13^, ^14^ The Million Women Study has also been linked to data from the National Cancer Intelligence Network (NCIN), providing additional information on diagnostic histology codes, and on tumour grade, for cases diagnosed in England. For this study, tumour histology and grade information from NCIN were used where available, to supplement the NHSCR data.

All participants gave written consent to follow‐up at recruitment. Ethical approval was granted by the Oxford and Anglia Multi‐Centre Research Ethics Committee (MREC 97/01).

### Exposure variables

Women in the study were asked at recruitment “Have you been sterilised (had your tubes tied)?,” and if so, their age at sterilization (used for analyses of the timing of sterilization).

### Outcome

The outcome of interest was ovarian cancer (C56 in ICD‐10).^12^ For histotype analyses, the outcome was split into five histological groups: serous (ICD‐O codes 8441–8442, 8451, 8460–8463, 9014), mucinous (ICD‐O codes 8470–8490), endometrioid (ICD‐O codes 8380–8381, 8560, 8570, 8933, 8950), clear cell (ICD‐O codes 8310, 8313) and other.

For some exploratory analyses, tumour types were analysed separately by grade (available for about 40% of all cases). Three‐tier grading systems had generally been used (including those outlined by FIGO (International Federation of Gynecology and Obstetrics),^15^ WHO (World Health Organization),^16^ and Silverberg^17^), and the few cases classified as “grade 4” were grouped together with “grade 3” tumours.

Serous tumours were divided into low‐grade tumours [defined here as borderline (ICD‐O codes 8442, 8451, 8462, 8463) or grade 1 serous tumours], and high‐grade serous carcinoma (defined here as grade ≥2 serous tumours). The assumptions we made in converting tumour grade information from the three‐tier (grade 1–3) to the two‐tier (low‐grade *vs*. high‐grade) system for serous carcinoma were in line with those used in other publications.^4^, ^18^, ^19^

Endometrioid tumours were divided into two, *i.e*., grade 1 or 2, and grade 3, as it has been suggested that some high‐grade (grade 3) endometrioid ovarian carcinomas might be the same as high‐grade serous carcinomas^20^ and should be considered with them.^4^

Clear cell tumours were not divided by grade, as all clear cell ovarian cancers are high‐grade/grade 3, by definition.^4^, ^20^, ^21^

Mucinous tumours were split into mucinous borderline tumours (ICD‐O codes 8472–8473) and mucinous carcinoma (ICD‐O codes 8470–8471 and 8480–8490); the mucinous carcinomas were not further subdivided by tumour grade, as numbers were small.

### Statistical analysis

Women were excluded from the analyses if: (*i*) they had been diagnosed with any invasive cancer other than nonmelanoma skin cancer (ICD‐10 code C44) prior to recruitment (*n* = 66,221), (*ii*) they reported at recruitment having had both ovaries removed (bilateral oophorectomy), or if they were unsure whether they had or not (*n* = 170,769), or (*iii*) they had missing data on tubal ligation (*n* = 15,712). The remaining women (*N* = 1,132,914) contributed person‐years from the date of recruitment into the study until the date of registration for ovarian cancer, the date of death, or last date of follow‐up (December 31, 2013)—whichever was soonest. Women were censored at diagnosis of any nonovarian cancer. For analyses exploring effects of the timing of tubal ligation, women were excluded if they had missing information on age at tubal ligation (*n* = 14,684). About 1% of participants had been lost to follow‐up and such women are censored at the date when they were lost, contributing person‐years until then.

Cox (proportional hazards) regression models were used to estimate hazard ratios [referred to as relative risks (RRs)] of developing ovarian cancer by tubal ligation status. Attained age was the underlying time variable. There was no evidence of significant violation of the proportional hazards assumption, as assessed by graphical methods and tests based on Schoenfeld residuals.

All analyses were stratified by geographical region (10 regions corresponding to the areas covered by the cancer registries), and further adjusted for parity (0, 1, 2, 3+), use of the oral contraceptive pill (never, ever), family history of breast cancer (no, yes), hysterectomy (no, yes), use of menopausal hormones (never, ever), body mass index (BMI) (<25 kg/m^2^, 25–29 kg/m^2^, 30+ kg m^−2^), smoking history (never, past, current) and quintiles of socioeconomic status (based on the Townsend deprivation index).^22^ All adjustment variables were as reported at recruitment. For adjustment variables, missing values were assigned to a separate category. Exposure information was either missing or reported as unknown for ≤6% of women for all potential confounders. Sensitivity analyses were conducted excluding all women with missing data on covariates within the model.

Other factors (including alcohol consumption, physical activity and age at menarche) were explored as potential confounders, but were not included in the final model as their inclusion made no appreciable difference to the main estimate of effect.

Tests of heterogeneity in the relationship between tubal ligation and ovarian cancer risk by histotype were performed using a competing risks approach.^23^

To explore possible effect modification of the main relationship between tubal ligation and ovarian cancer, multiplicative interaction terms were modelled between tubal ligation and each potential confounder in turn. Likelihood ratio tests were used to compare the models with versus without the interaction terms.

Analyses were also conducted to explore possible effects of age and time with tubal ligation, including age at, time since and calendar year of tubal ligation, and whether the tubal ligation was in the same year as the last birth or afterward. All analyses of the timing of tubal ligation were restricted to parous women, as there were very few nulliparous women who had had a tubal ligation. Where comparisons are made between more than two exposure categories, group‐specific confidence intervals for the log risk in each group were calculated, allowing comparisons to be made between any two categories, even if neither is the reference group.^24^ Conventional 95% confidence intervals (CI) are given in the text.

Analyses were performed in Stata‐14.^25^ Tests of statistical significance were two‐sided. Figures were drawn in R using Matthew Arnold and Paul Sherliker's “Jasper” package.

## Results

A total of 1,132,914 women, mean age at recruitment 56.1 [standard deviation (SD) 4.8] years, were included in the analyses. At recruitment, 246,048 (22%) reported having had a tubal ligation, at median age 35 [interquartile range (IQR) 31–38], and median year 1978 (IQR 1973–1982). Women who reported a previous tubal ligation were more likely to be parous, to have used the oral contraceptive pill and menopausal hormones, to have had a hysterectomy, to be a current smoker, and to live in areas in the lower third of socioeconomic status, compared to women without tubal ligation (Table 1).

**Table 1:** Characteristics of the study population at recruitment, and details of follow‐up, by tubal ligation status

	Tubal ligation		
Characteristics	No	Yes	All women
Number of women	886,866	246,048	1,132,914
Mean (SD) age at recruitment (years)	56.3 (4.9)	55.3 (4.3)	56.1 (4.8)
Socioeconomic status, lower third, % (*n*)	30.5 (268,314)	38.0 (92,663)	32.1 (360,977)
Mean age at menarche (SD)	13.0 (1.6)	13.0 (1.6)	13.0 (1.6)
Nulliparous, % (*n*)	13.3 (117,319)	2.6 (6,352)	10.9 (123,671)
Ever use of oral contraceptive pill, % (*n*)	57.0 (501,857)	70.3 (171,546)	59.9 (673,403)
Ever use of menopausal hormones, % (*n*)	44.5 (390,518)	54.9 (133,529)	46.7 (524,047)
Hysterectomy, % (*n*)	14.2 (126,054)	19.4 (47,579)	15.4 (173,633)
Mean age at natural menopause (SD)	49.3 (4.2)	48.7 (4.5)	49.1 (4.3)
Family history of breast cancer, % (*n*)	9.8 (82,101)	10.0 (22,781)	9.9 (104,882)
Mean (SD) body mass index (kg/m^2^)	26.0 (4.6)	26.6 (4.8)	26.1 (4.7)
Current smoker, % (*n*)	18.4 (153,773)	26.4 (61,264)	20.1 (215,037)
Strenuous exercise ≥once/week, % (*n*)	39.8 (340,538)	37.7 (89,266)	39.3 (429,804)
Alcohol intake, ≥7 units/week, % (*n*)	23.6 (207,870)	25.5 (62,130)	24.0 (270,000)
**Follow‐up for incident ovarian cancer**			
Woman‐years of follow‐up for incidence (100,000s)	122.6	33.9	156.5
Mean follow‐up time per woman (SD)	13.8 (3.4)	13.8 (3.3)	13.8 (3.4)
Number of incident ovarian cancers	6,693	1,342	8,035

Means and percentages are calculated excluding missing values for the variable of interest.

*n*: number of women. SD: standard deviation.

The women were followed up for incident ovarian cancer over 15.6 million person‐years, with a mean duration of follow‐up of 13.8 (SD 3.4) years per woman. During this period, 8,035 incident ovarian cancers were registered, of which 3,515 (44%) were serous; 836 (10%) were mucinous; 690 (9%) were endometrioid; 401 (5%) were clear cell and 2,593 (32%) were of other histological types (mostly unspecified epithelial tumours). The mean age at diagnosis of ovarian cancer was 65.1 years (SD 6.4).

The relative risk of ovarian cancer amongst women with tubal ligation compared to those without was 0.80 (95% CI: 0.76–0.85, *p* < 0.001), after adjustment for age, region, parity, family history of breast cancer, hysterectomy, socioeconomic status, body‐mass index, smoking and use of contraceptive or menopausal hormones.

**Figure 1:**
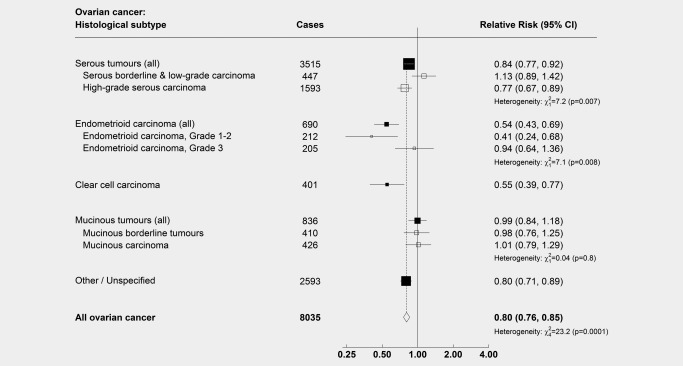
**Relative risk of subtypes of ovarian cancer in women with versus without a history of tubal ligation**. *N* = 1,132,914. Results show relative risks (hazard ratios) of ovarian cancer amongst women with a history of tubal ligation compared to women without a history of tubal ligation, by histological subtype. Analyses are adjusted for age, region, parity, family history of breast cancer, hysterectomy, use of the oral contraceptive pill and menopausal hormones, body mass index, smoking and socioeconomic status. Serous tumours have been split into low‐grade (serous borderline tumours and low‐grade serous carcinoma, here defined as grade 1) and high‐grade (serous carcinoma of grade ≥2). Endometrioid tumours have been split into low‐grade (here defined as grade 1–2) and high‐grade (grade 3); see main text for discussion of rationale for this. Note: The numbers of grade‐specific serous and endometrioid tumours do not sum to the total numbers of serous and endometrioid tumours, as information on tumour grade was missing for 1,475 serous carcinomas and 273 endometrioid carcinomas.

There was strong evidence of heterogeneity by histotype (Fig. 1; heterogeneity, *p* = 0.0001). Tubal ligation was associated with almost a halving in the risk of endometrioid (RR: 0.54, 95% CI: 0.43–0.69) and clear cell tumours (RR: 0.55, 95% CI: 0.39–0.77), and a lesser but still significant reduction in the risk of serous tumours (RR: 0.84, 95% CI: 0.77–0.92), but there was no significant reduction in the risk of mucinous tumours (RR: 0.99, 95% CI: 0.84–1.18).

When we divided serous tumours into low‐grade serous tumours (serous borderline tumours and low‐grade serous carcinoma) and high‐grade serous carcinoma, risks differed significantly (heterogeneity, *p* = 0.007). There was strong evidence that tubal ligation was associated with a reduction in the risk of high‐grade serous carcinoma (*n* = 1,593, RR: 0.77, 95% CI: 0.67–0.89), but not of low‐grade serous tumours (*n* = 447, RR: 1.13, 95% CI: 0.89–1.42).

In view of suggestions that some tumours classified as high‐grade endometrioid tumours might have been mis‐diagnosed high‐grade serous tumours,^4^ we conducted an exploratory analysis, splitting endometrioid tumours into low‐grade carcinoma, and high‐grade carcinoma. There was again heterogeneity in the risks (*p* = 0.008), with tubal ligation associated with a greater reduction in the risk of low‐grade (*n* = 212, RR: 0.41, 95% CI: 0.24–0.68), than of high‐grade endometrioid carcinoma (*n* = 205, RR: 0.94, 95% CI: 0.64–1.36).

When mucinous tumours were split into borderline and fully malignant subtypes, there was little or no association between tubal ligation and the risk of either mucinous borderline tumours (*n* = 410, RR: 0.98, 95% CI: 0.76–1.25) or fully malignant mucinous carcinoma (*n* = 426, RR: 1.01, 95% CI: 0.79–1.29).

A sensitivity analysis excluding all women with missing values in any of the adjustment variables showed that the association between tubal ligation and ovarian cancer, and the variation by histotype, was not appreciably changed (data not shown).

**Figure 2:**
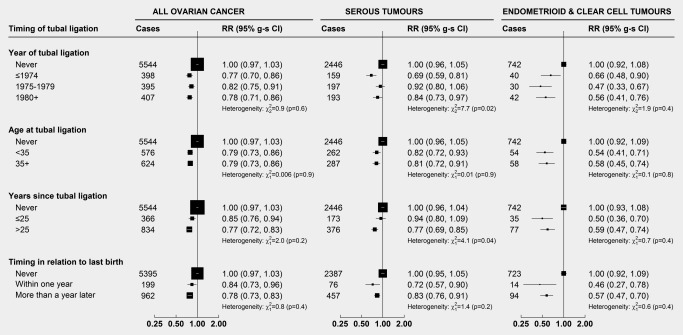
**Relative risk of ovarian cancer in relation to the timing of tubal ligation (amongst parous women only)**. Results show relative risks (hazard ratios) of ovarian cancer by the timing of tubal ligation, with 95% group‐specific confidence intervals (95% g‐s CI). Each timing analysis was a separate model. For each analysis, we excluded nulliparous women, and sterilized women if their age at tubal ligation was unknown [993,166 women (6,744 cases) included in analyses]. For the analysis of tubal ligation at versus after the last birth, women with an unknown age at last birth were also excluded [967,166 women (6,556 cases) included in analyses]. Analyses are adjusted for age, region, parity, family history of breast cancer, hysterectomy, use of the oral contraceptive pill and menopausal hormones, body mass index, smoking and socioeconomic status. Heterogeneity tests are among women with a tubal ligation only.

There was little or no variation in risk either by age at, year of, or years since tubal ligation, or whether tubal ligation was performed in the same year as the last birth or subsequently, for ovarian cancer overall, and for endometrioid and clear cell tumours (Fig. 2). Compared to women without tubal ligation, for serous tumours there was some suggestion of a greater reduction in risk with tubal ligations performed ≤1974 (RR: 0.69, 95% CI: 0.58–0.81), than with those performed in 1975–1979 (RR: 0.92, 95% CI: 0.80–1.07), or those performed in 1980 or later (RR: 0.84, 95% CI: 0.73–0.98), heterogeneity: *p* = 0.02. There was also some suggestion of a greater reduction in risk for women with a time since tubal ligation of >25 years (RR: 0.77, 95% CI: 0.69–0.86) than for ≤25 years since tubal ligation (RR: 0.94, 95% CI: 0.80–1.10), heterogeneity: *p* = 0.04.

**Figure 3:**
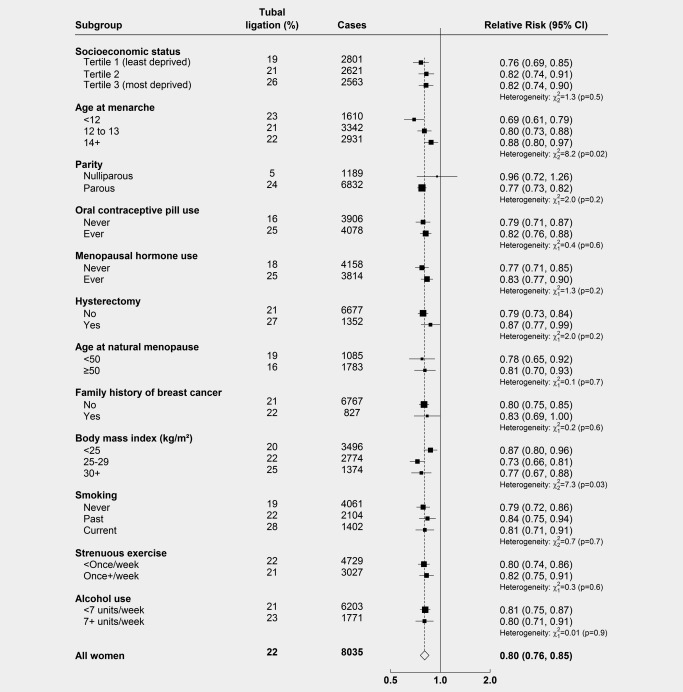
**Relative risk of ovarian cancer in women with versus without a history of tubal ligation, by subgroup**. Results show the relative risks (hazard ratios) of ovarian cancer in women with versus without tubal ligation, by various other factors. The results for each subgroup are from separate models, restricted to women with no missing information on that variable, and thus total numbers of participants and cases will differ. Analyses are adjusted for age, region, parity, family history of breast cancer, hysterectomy, use of the oral contraceptive pill or menopausal hormones, body mass index, smoking and socioeconomic status, as appropriate.

There was no significant variation in the association between tubal ligation and ovarian cancer with parity, use of contraceptive or menopausal hormones, hysterectomy, age at natural menopause, family history of breast cancer, smoking, socioeconomic status, frequency of strenuous exercise or alcohol intake (Fig. 3). There was some weak evidence of heterogeneity by body mass index and age at menarche, although this may well be due to chance, as multiple statistical tests were performed.

## Discussion

In a large prospective cohort study with 8,035 incident cases of ovarian cancer, we found strong evidence that the risk of ovarian cancer associated with tubal ligation varied by tumour histotype. We found a modest reduction in risk of the most common histotype, serous tumours, as had been previously reported.^7^, ^8^, ^9^ However, when we split serous tumours into low‐grade serous tumours and high‐grade serous carcinoma, we found significant differences, with a 20% reduction in risk of high‐grade serous carcinoma, but no reduction in risk of low‐grade serous tumours. This is the first study to report a significant difference between the risks of low‐grade versus high‐grade serous ovarian tumours associated with tubal ligation.

We found about a halving of the risk of endometrioid and of clear cell tumours associated with tubal ligation. Others have reported similar findings for endometrioid tumours.^7^, ^8^, ^9^ Reported findings for clear cell tumours are mixed, perhaps because it is relatively uncommon. We found little or no association between tubal ligation and the risk of mucinous ovarian cancer, in keeping with some reports,^7^, ^8^ though not others.^9^

New hypotheses about the origins of ovarian cancer have provoked a reconsideration of possible mechanisms underlying the reduced risk of ovarian cancer with tubal ligation.^7^, ^9^, ^26^, ^27^ The significant heterogeneity seen here between low‐grade versus high‐grade serous tumours is consistent with the hypothesis that the two tumour subtypes develop along distinct pathways.

High‐grade serous ovarian cancer is hypothesised to arise from precursor lesions within the fallopian tubal epithelium, particularly in the fimbrial end adjacent to the ovary, and subsequently seed to the ovary. There is considerable evidence for this, including several studies showing putative precursor lesions within the fallopian tubes (such as focal areas of epithelium featuring mutations in the tumour‐suppressor gene *TP53* or aberrant expression of its protein p53, and/or areas of dysplasia or intraepithelial serous carcinoma), not only in prophylactic salpingoophorectomy specimens from women with an increased genetic risk of ovarian cancer, and from women with known high‐grade serous ovarian cancer,^28^, ^29^, ^30^, ^31^, ^32^, ^33^, ^34^ but also in salpingoophorectomy specimens from women not known to be at increased genetic risk, in some cases with accompanying incidental invasive high‐grade serous carcinoma.^35^, ^36^, ^37^ The putative precursor lesions in many cases show molecular changes that are similar or identical to those seen in co‐existing high‐grade serous ovarian cancer, particularly in terms of *TP53* mutations^33^ (mutations in the gene for p53 being characteristic and ubiquitous in high‐grade serous ovarian cancer^38^).

The origins of low‐grade serous tumours are considerably more uncertain. In many cases, there appears to be a step‐wise progression from ovarian epithelial inclusion cysts, through benign cystadenomas and serous borderline tumours, to low‐grade serous carcinoma. Evidence for this includes the frequent co‐existence of serous borderline tumours with low‐grade serous carcinomas,^18^ and similar molecular changes: both serous borderline tumours and low‐grade serous carcinomas commonly feature mutations in *BRAF* or *KRAS*,^39^ but rarely in *TP53*,^40^ in contrast to high‐grade serous carcinoma. It has been suggested that some low‐grade serous tumours may also originate indirectly from tubal epithelial cells (perhaps from benign or hyperplastic tubal epithelial cells which become trapped in the ovary as epithelial inclusion cysts at the time of ovulation, possibly at a young age),^41^, ^42^ but the evidence for this is much less established than that for the tubal origins of high‐grade serous carcinoma.

Surgical ligation of the fallopian tube might reduce the risk of high‐grade serous ovarian cancer, either by physically obstructing the passage of tubal cells toward the ovary, or by collateral damage to the local tubal blood supply, with potential subsequent effects on the tubal epithelium. In addition, some forms of tubal sterilization involve removal of some or all of the tube, and/or disruption of the distal end of the tube—which might have been more common in the past, and could possibly explain the significantly greater risk reduction found for tubal ligations done before than after 1974.

The apparent lack of an effect of tubal ligation for low‐grade serous tumours is consistent with the hypothesis that they have a different origin to high‐grade serous ovarian cancer. However, it is not entirely incompatible with a possible tubal origin for low‐grade serous tumours (*e.g*., if the precursor cells of origin had already been attached to the ovary before the tubal ligation occurred).

Some endometrioid and clear cell tumours are thought to develop from endometriosis,^3^, ^4^ and one might speculate that the almost halving of risk seen with tubal ligation could be due to the blocking of retrograde menstruation through the fallopian tubes to the ovaries and pelvis. It could also reflect the blocking of the transport of carcinogens up the tubes to the ovaries and pelvis. For any mechanism that involved the blockage of menstruation or the passage of carcinogens up the tube, one might expect the association with tubal ligation to vary by timing (*e.g*., a greater reduction in risk for tubal ligation performed at a younger age, or longer ago). However, we found no suggestion of any association with the timing of tubal ligation for ovarian cancer overall or for endometrioid and clear cell tumours, and only limited evidence of an association with the year of, and years since, tubal ligation for serous tumours. These findings are in keeping with most, but not all, previous studies.^7^, ^8^, ^9^

Our study has several strengths, in addition to the large sample size and number of cases. In particular, the prospective collection of exposure data helped to prevent differential recall of tubal ligation or other factors amongst women with and without cancer. The use of routinely collected national data for follow‐up and ascertainment of incident ovarian cancer resulted in few participants being lost to follow‐up. The prospective study design also facilitated the involvement of women with more aggressive tumour types, who might not be included in retrospective studies, as there can be a substantial time lag between cancer diagnosis and recruitment to a retrospective study. The high prevalence of tubal ligation (22%) was also an advantage in terms of statistical power.

One potential weakness of our study was that we did not undertake central histopathological review of our cases, for standardization and incorporation of the latest diagnostic criteria. The distribution of histotypes amongst our cases was consistent with those from other population‐based studies, with serous tumours accounting for around 65% of those of the four main histotypes, endometrioid tumours for 13%, mucinous 15%, and clear cell 7% (by way of comparison, the corresponding percentages from the EPIC cohort are: 67% serous, 13% endometrioid, 14% mucinous, and 6% clear cell^43^). This distribution is slightly different to that reported by two hospital‐based studies of ovarian cancer cases, in both of which mucinous tumours accounted for only around 3% of cases^44^, ^45^; this may reflect differences in the study populations (North America *vs*. Europe), or historical differences in diagnostic criteria (*e.g*., mucinous tumours in our cohort might include some metastases from the gastrointestinal tract).

About 32% of ovarian cancers in our study were not recorded as being of one of the four main histotypes (serous, endometrioid, clear cell or mucinous). Of these, 87% were epithelial tumours of other, mixed, or unspecified type (mostly unspecified adenocarcinoma, ICD‐O code 8140, or unspecified carcinoma, ICD‐O code 8010), and 10% were unspecified malignant tumours (ICD‐O code 8000). Given that high‐grade serous carcinoma is the most common histotype of ovarian cancer, it seems likely that many of the tumours listed as unspecified carcinoma/adenocarcinoma are in fact high‐grade serous tumours. This would be supported by our observation that tubal ligation is associated with a similar reduction in risk of other/unspecified ovarian cancer (RR: 0.80, 95% CI: 0.71–0.89) as of high‐grade serous carcinoma (RR: 0.77, 95% CI: 0.67–0.89).

Tumour histology may have been classified in somewhat different ways by different pathologists. Any such misclassification would tend to blur differences by tumour histotype, yet distinctly heterogeneous risks were found. This argues strongly for causality, as it confirms that the variation in risk associated with tubal ligation is not just due to confounding, and strengthens hypotheses that different histotypes of ovarian cancer have different causes.

## Million Women Study Collaborators

**Million Women Study Co‐ordinating Centre staff**: Hayley Abbiss, Simon Abbott, Rupert Alison, Naomi Allen, Miranda Armstrong, Krys Baker, Angela Balkwill, Emily Banks, Isobel Barnes, Valerie Beral, Judith Black, Roger Blanks, Kathryn Bradbury, Anna Brown, Benjamin Cairns, Karen Canfell, Dexter Canoy, Andrew Chadwick, Barbara Crossley, Francesca Crowe, Dave Ewart, Sarah Ewart, Lee Fletcher, Sarah Floud, Toral Gathani, Laura Gerrard, Adrian Goodill, Jane Green, Lynden Guiver, Michal Hozak, Isobel Lingard, Sau Wan Kan, Oksana Kirichek, Nicky Langston, Bette Liu, Kath Moser, Kirstin Pirie, Gillian Reeves, Keith Shaw, Emma Sherman, Helena Strange, Sian Sweetland, Sarah Tipper, Ruth Travis, Lyndsey Trickett, Lucy Wright, Owen Yang, Heather Young.

**Million Women Study Advisory Committee**: Emily Banks, Valerie Beral, Lucy Carpenter, Carol Dezateux, Jane Green, Julietta Patnick, Richard Peto, Cathie Sudlow.

**The NHS Breast Screening Centres which took part in the recruitment of participants were**: Avon, Aylesbury, Barnsley, Basingstoke, Bedfordshire and Hertfordshire, Cambridge and Huntingdon, Chelmsford and Colchester, Chester, Cornwall, Crewe, Cumbria, Doncaster, Dorset, East Berkshire, East Cheshire, East Devon, East of Scotland, East Suffolk, East Sussex, Gateshead, Gloucestershire, Great Yarmouth, Hereford and Worcester, Kent, Kings Lynn, Leicestershire, Liverpool, Manchester, Milton Keynes, Newcastle, North Birmingham, North East Scotland, North Lancashire, North Middlesex, North Nottingham, North of Scotland, North Tees, North Yorkshire, Nottingham, Oxford, Portsmouth, Rotherham, Sheffield, Shropshire, Somerset, South Birmingham, South East Scotland, South East Staffordshire, South Derbyshire, South Essex, South Lancashire, South West Scotland, Surrey, Warrington Halton St Helens and Knowsley, Warwickshire Solihull and Coventry, West Berkshire, West Devon, West London, West Suffolk, West Sussex, Wiltshire, Winchester, Wirral, Wycombe.
